# Hepatitis B virus infection as a risk factor for chronic kidney disease: a systematic review and meta-analysis

**DOI:** 10.1186/s12879-024-09546-z

**Published:** 2024-06-22

**Authors:** Danjing Chen, Rong Yu, Shuo Yin, Wenxin Qiu, Jiangwang Fang, Xian-e Peng

**Affiliations:** 1 Department of Epidemiology and Health Statistics, Fujian Provincial Key Laboratory of Environment Factors and Cancer, School of Public Health, Fujian Medical University, Fuzhou, 350122 People’s Republic of China; 2 Department of Epidemiology and Health Statistics, Key Laboratory of Gastrointestinal Cancer, School of Basic Medical Sciences, Fujian Medical University, Ministry of Education, Fujian Medical University, Xuefu North Road 1st, Shangjie Town, Minhou Country, Fuzhou, Fujian 350108 China

**Keywords:** Hepatitis B virus, Chronic kidney disease, Meta-analysis, Risk

## Abstract

**Background:**

Currently, several studies have observed that chronic hepatitis B virus infection is associated with the pathogenesis of kidney disease. However, the extent of the correlation between hepatitis B virus infection and the chronic kidney disease risk remains controversial.

**Methods:**

In the present study, we searched all eligible literature in seven databases in English and Chinese. The random effects model was used to conduct a meta-analysis. Quality of included studies was assessed using the Newcastle-Ottawa Quality Scale.

**Results:**

In this analysis, a total of 31 studies reporting the association between hepatitis B virus infection and chronic kidney disease risk were included. The results showed a significant positive association between hepatitis B virus infection and the risk of chronic kidney disease (pooled *OR*, 1.20; 95% *CI*, 1.12–1.29), which means that hepatitis B virus increases the risk of developing chronic kidney disease.

**Conclusion:**

This study found that hepatitis B virus infection was associated with a significantly increased risk of chronic kidney disease. However, the current study still cannot directly determine this causal relationship. Thus, more comprehensive prospective longitudinal studies are needed in the future to provide further exploration and explanation of the association between hepatitis B virus and the risk of developing chronic kidney disease.

**Supplementary Information:**

The online version contains supplementary material available at 10.1186/s12879-024-09546-z.

## Introduction

Chronic kidney disease (CKD) is the primary non-infectious disease associated with high morbidity and mortality and is commonly defined as persistent urinary abnormalities, structural abnormalities, or impaired renal excretory function [1, 2]. When diagnosed with CKD, kidney function gradually declines and progresses to end-stage renal disease (ESRD) with irreversible damage [3]. It is estimated that patients with CKD account for more than 10% of the world’s population, and the prevalence increases with age [4, 5]. In addition, the researchers found that both morbidity and mortality from CKD have risen dramatically over the past 30 years, and that this upward trend will continue through 2029 [6]. Therefore, CKD is considered a growing global public health problem.

Currently, about 296 million people worldwide are infected with hepatitis B virus (HBV), which is the main cause of cirrhosis and liver cancer [7]. Besides the effects on the liver, several studies have found that chronic HBV infection is associated with the pathogenesis of kidney diseases such as polyarteritis nodosa (PAN) catheterization and glomerulonephritis (GN) [8]. Recently, an increasing number of studies have been conducted on the relationship between HBV infection and CKD. However, the extent of the association between the two remains controversial. A large U.S. cohort study found that HBV infection was associated with an increased risk of developing CKD and ESRD [9]. However, a cross sectional study based on a Chinese population did not find any direct relationship between HBV infection and the risk of developing CKD [10]. Recently, a meta-analysis showed that HBV infection is related to an increased risk of CKD in the general adult population [11]. The recently publication on the relationship between HBV and CKD provides an opportunity to assess again the association between HBV and CKD, which may provide additional scientific evidence [12–14]. Therefore, in this study, we assessed the association between HBV and the risk of CKD prevalence in the general adult population through a meta-analysis of observational studies.

## Materials and methods

### Literature search strategy

All relevant studies up to March 20, 2023 were searched all eligible literature in seven databases in English and Chinese, including Chinese National Knowledge Infrastructure (CNKI), China Science, Wanfang and Technology Journal (VIP), PubMed, Web of Science, Embase databases and Cochrane Library. The search terms included “hepatitis B virus infection”, “chronic hepatitis B”, “HBV”, “chronic kidney disease “, “CKD”, and “chronic renal insufficiency”. The search formulas have been adjusted to the requirements of each database separately. Besides the above search methods, manual searches were performed for references to reviews and original articles. Supplementary Material 1 shows in detail the specific search formulas used for each database.

### Study selection

There were no language limitations for studies included in the analysis, but review articles, abstracts, reviews, letters, and articles without complete text or valid data were excluded. When more than one study reported similar data, the most recent study was included in this analysis. In addition, for inclusion, the following requirements were met: (a) the type of study design was a cohort study, case-control study, or cross-sectional study; (b) HBV infection is defined as detection of HBsAg in serum and/or HBV DNA by PCR [15]; (c) the study outcome was the incidence or prevalence of CKD (glomerular filtration rate (GFR) < 60 mL/min/1.73 m^2^ or albuminuria ≥ 30 mg/24 hours) or ESRD or composite renal outcome due to CKD [1]; (d) an adjusted risk estimates or sufficient data to calculate the above metrics.

### Data extraction

Information was independently extracted from the retrieved literature by two authors according to the inclusion exclusion criteria. When disagreements arose, they were analyzed and resolved by a third researcher. Information extracted from the literature included mainly (a) the sample size of the study, (b) details of the study design, (c) patient characteristics, (d) outcome indicators as defined above.

### Quality assessment

The quality of the included 20 case-control studies and cohort studies was assessed using the Newcastle-Ottawa Quality Scale (NOS) [16]. The NOS scoring criteria included three main components: selection of study subjects, comparability between groups, and outcome/exposure assessment. Points were assigned when the information contained in the articles matched the scale description. Of these, those scoring below 4 were classified as low-quality studies, those scoring 5–6 as moderate-quality studies, and those scoring above 7 as high-quality studies. In addition, the quality of the 11 included cross-sectional studies was assessed according to the adapted NOS [17]. Studies with scores of 6–10, 4–5, or 0–3 were rated as high quality, moderate quality, and low quality, respectively. Only articles rated as moderate and high quality were included in the meta-analysis.

### Statistical analysis

A meta-analysis of the included literature was performed using Stata 17.0 software. Odds risks (*OR*) or hazard ratios (*HR*) and their 95% confidence intervals (*CI*) were used to estimate effect sizes. Meanwhile, the *I*^2^ statistic and *Q* test were used to assess possible heterogeneity between different study results. Included studies were considered to have large heterogeneity when *I*^*2*^⩾50% or *P* < 0.05. When study heterogeneity existed, a random effects model was used to calculate pooled effect sizes. Conversely, a fixed-effects model was used. Besides, when there was significant heterogeneity across studies, meta-regression and subgroup analysis were used to explore the sources of heterogeneity. Also, sensitivity analysis was performed using the one-by-one exclusion method. Begg’s test, Egger’s test, and funnel plot were used to assess the potential publication bias of the included literature. All *P*-values were obtained in a two-sided test.

## Results

### Study selection and study characteristics

**Fig. 1:**
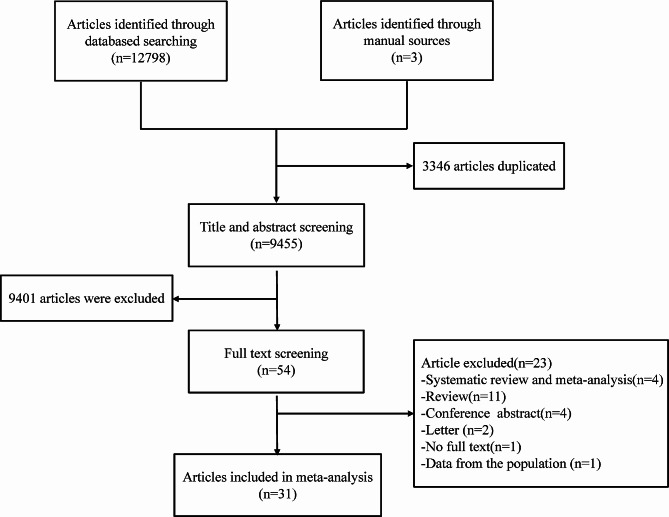
Flowchart of the selection of studies for inclusion in the meta-analysis

A total of 12,801 studies were collected by a search of seven Chinese and English databases and a manual search of references. The retrieved articles were managed using EndNote software. The literature was selected based on inclusion and exclusion criteria, and a total of 31 studies were eligible [10, 12–14, 18–44], which included three manually searched articles. Among them, studies by Hwang JC et al. [25] and Tartof SY et al. [37] were included for the first time in 2019 [45], whereas Chen YC et al. [19] were included for the first time in 2020 [11] in the systematic review and meta-analysis. Finally, 11 of the included articles were cross-sectional studies, 16 were cohort studies, and the remaining 4 were case-control studies. Figure 1 shows the specific process of literature screening. The general characteristics of the final included studies are shown in Table 1.

**Table 1:** Main characteristics of studies included in the review that reported the relationship between HBV and CKD risk

Author	Reference Year	Study Area	Type of Study	Patients (*n*)	HBV Positive (%)	Outcome	*OR*/*HR*	95%*CI*
Cai J	2012	China	Cross-sectional	6854	4.8	CKD	0.77	0.52–1.19
Chen YC	2015	China (Taiwan)	Cohort	88980	20	CKD	2.58	1.95–3.42
Du Y	2019	China	Cohort	2969502	5.57	CKD	1.55	1.44–1.67
Geng XX	2020	the U.S.	Cohort	353370	NA	CKD	1.06	1.00-1.12
Hong Y	2018	Korea	Cohort	299913	2.5	CKD	1.11	1.03–1.21
Ishizaka N	2008	Japan	Cross-sectional	12535	1.04	CKD	0.49	0.30–0.81
Kim SE	2018	Korea	Case-control	50240	NA	CKD	1.23	1.04–1.45
Kong XL	2016	China	Cohort	4329	8.10	CKD	1.12	0.65–1.95
Lee JJ	2010	China (Taiwan)	Cross-sectional	54966	9.9	CKD	1.04	0.96–1.14
Lin MY	2012	China (Taiwan)	Cross-sectional	3352	12.4	CKD	1.35	1.03–1.77
Lin S	2020	China	Cross-sectional	32578	14.5	CKD	1.39	1.06–1.81
Liu Y	2021	China	Case-control	642	62.77	CKD	2.10	1.13–3.91
Mocroft A	2012	Australia, United Kingdom, Denmark	Cohort	3441	3.3	Composite Outcome	2.26	1.15–4.44
Senghore T	2013	China (Taiwan)	Cross-sectional	7745	7.36	CKD	1.11	0.86–1.43
Si J	2018	China	Cohort	469459	3.17	Composite Outcome	1.37	1.18–1.60
Zeng Q	2014	China	Cross-sectional	15549	5.05	CKD	1.23	0.83–1.80
Zhang H	2019	China	Cross-sectional	2435	4.8	CKD	0.73	0.44–1.19
Zhang L	2008	China	Cross-sectional	13925	1.1	CKD	0.97	0.87–1.06
Cheng AY	2006	China	Cohort	2838	10.08	Composite Outcome	4.53	1.11–18.58
Du Y	2017	China	Cross-sectional	3091379	5.64	CKD	1.27	1.24–1.31
Fang J	2018	China	Cohort	177	47.46	Composite Outcome	4.03	0.98–13.20
Hwang JC	2016	China (Taiwan)	Cohort	19574	5.48	ESRD	1.23	0.68–2.23
Lai TS	2017	China (Taiwan)	Cohort	13805	16.8	CKD	0.95	0.72–1.24
Lee JJ	2014	China (Taiwan)	Cohort	4185	7.4	ESRD	1.10	0.89–1.35
Nguyen MH	2019	the U.S.	Cohort	165594	26.15	Composite Outcome	1.09	1.02–1.17
Su SL	2015	China (Taiwan)	Case-control	10,463	4.06	CKD	1.25	1.03–1.52
Vu V	2019	the U.S.	Case-control	580	50	CKD	1.25	0.68–2.31
Chen YC	2018	China (Taiwan)	Cohort	14580	3.2	ESRD	1.67	1.40–1.98
Huang JF	2006	China	Cross-sectional	9934	13.1	CKD	0.94	0.75–1.17
Lo MK	2004	China (Hongkong)	Cohort	97	16.13	ESRD	1.00	0.50–1.50
Tartof SY	2018	Americas	Cohort	152708	0.3	ESRD	1.17	0.78–1.75

### Quality assessment

According to the NOS quality assessment of the included literature, a total of 13 case-control or cohort studies and 11 cross-sectional studies were considered to be of high quality, and the other 7 included cohort studies were of moderate quality. The proportion of high-quality studies is 77.4% (24/31). Details used to rate the quality of the studies are shown in Supplementary Tables 1 and 2.

### Meta-analysis

**Fig. 2:**
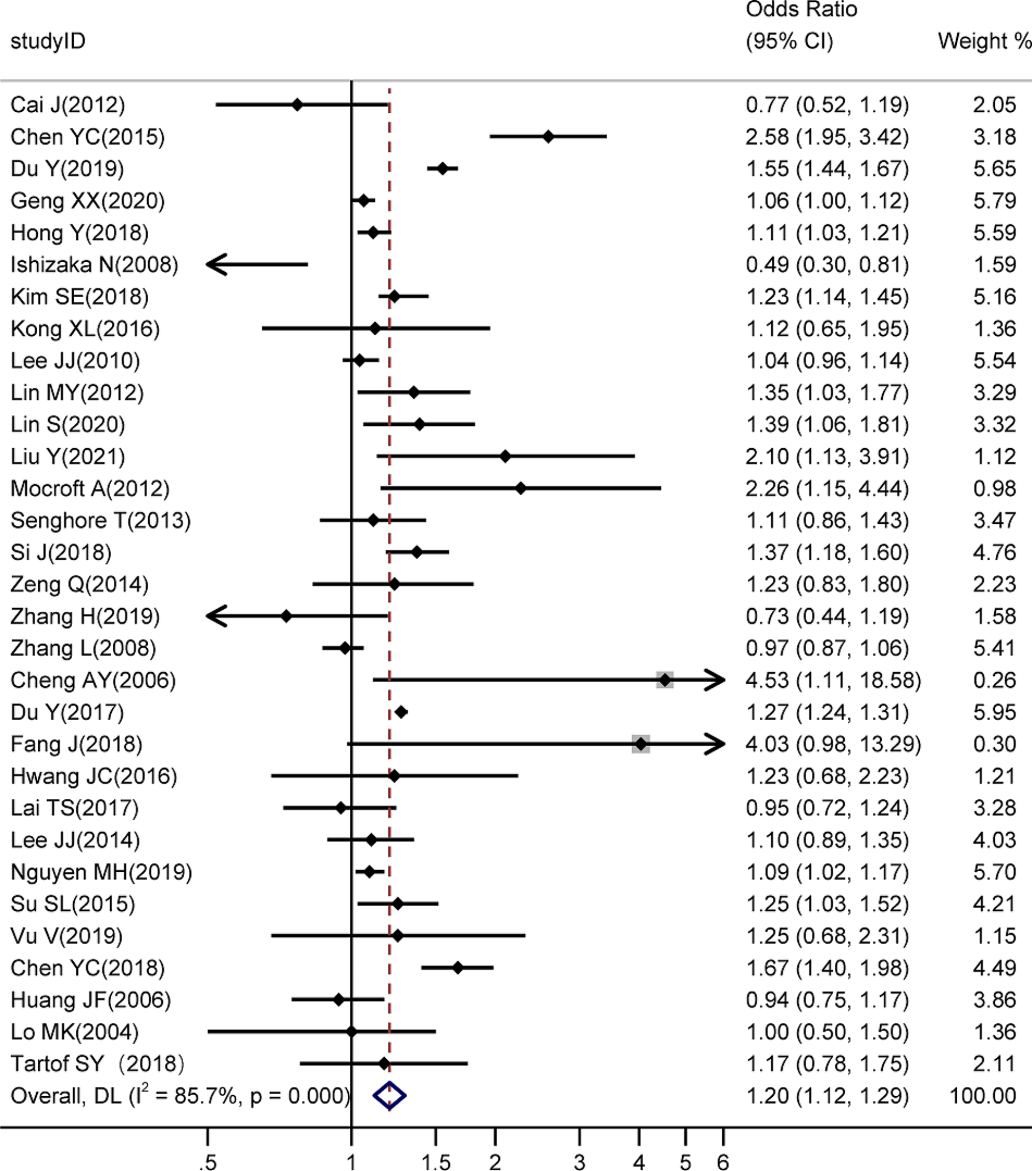
Forest plot of association meta-analysis of HBV and CKD risk

A random-effects model was used to perform a meta-analysis of the 31 included studies reporting the association between HBV and CKD risk. As the result is shown in Fig. 2, there was a significant positive association between HBV infection and the risk of CKD (pooled *OR*, 1.20; 95% *CI*, 1.12–1.29), which means that HBV infection increases the risk of developing CKD. Furthermore, a large statistical heterogeneity was found in this meta-analysis (*I*^*2*^ = 85.7%, *P* < 0.001).

Meta-regression analysis was performed on five factors including type of study, region, reference year, study outcome, and sample size of the included articles to explore sources of heterogeneity. The result is shown in Table 2, and no heterogeneity was generated by including these five variables in the regression model simultaneously. In addition, subgroup analyses were conducted on the five factors mentioned above, and the results, as shown in Supplementary Figs. 1–5, did not reveal a source of heterogeneity.

**Table 2:** Meta regression analysis of HBV and risk of CKD

Subgroup variable	t	*P*	95%CI
Type of Study	-1.27	0.216	-0.25, 0.06
Area of Study	-1.25	0.224	-0.57, 0.14
Study Outcome	0.55	0.587	-0.15, 0.26
Reference year	1.13	0.268	-0.09, 0.30
Sample size	-0.27	0.788	-0.30, 0.23

### Sensitivity analysis and publication bias

**Fig. 3:**
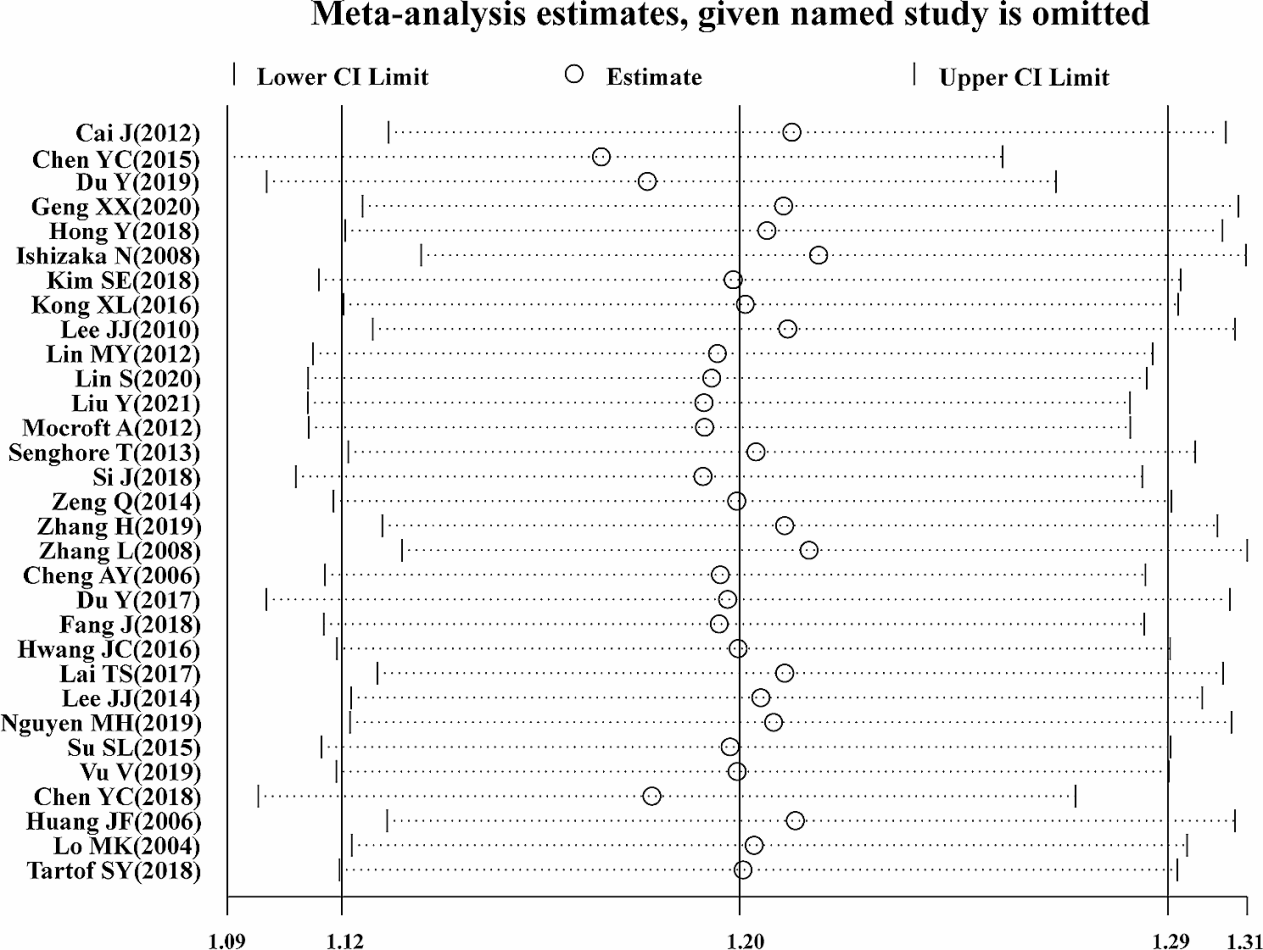
Sensitivity analysis of the association between HBV and CKD risk

**Fig. 4:**
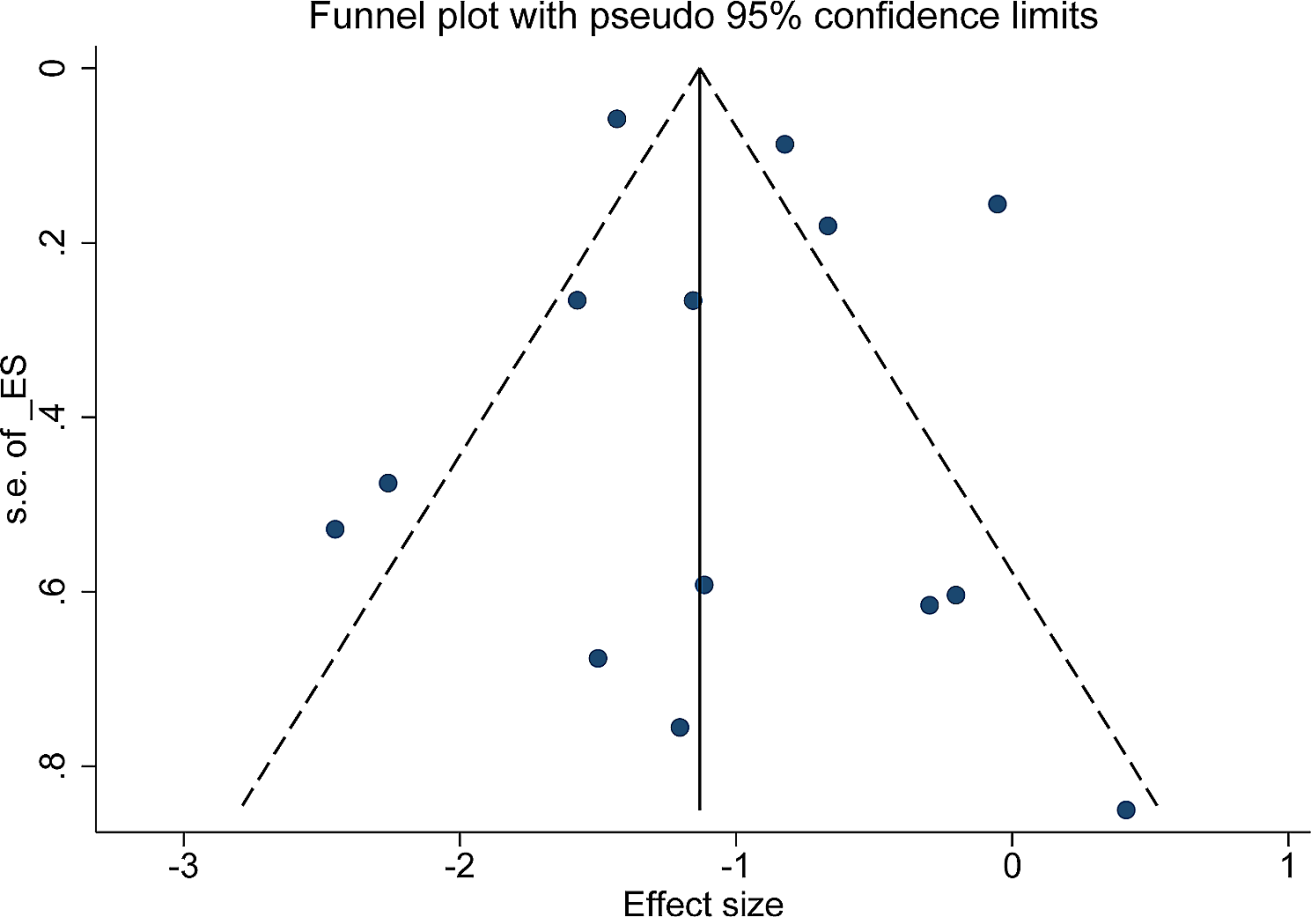
Funnel plot of the association between HBV and CKD risk

As shown in Fig. 3, a sensitivity analysis of the included studies was performed using a case-by-case exclusion method to evaluate the impact of individual studies on the newly generated pooled *OR*. As the results showed, the results of the meta-analysis were comparatively stable after excluding any of the studies, and ranged from 1.17 (95% *CI*, 1.09–1.26) to 1.22 (95% *CI*, 1.13–1.31). The *P* values of the regression tests of Egger and Begg used to test for publication bias were 0.862 and 0.139, which were consistent with the results suggested by the funnel plot (Fig. 4), and there was no publication bias in this study.

## Discussion

Over the past few decades, a strong link between HBV and kidney disease has been known to exist [46, 47]. However, controversy remains regarding the relationship between HBV infection and CKD risk. This study summarized and pooled the relevant existing studies to perform a meta-analysis of the risk of CKD in the adult general population infected with HBV. The results showed that people infected with HBV had a higher risk of developing CKD compared to those who were not infected with HBV (pooled *OR*, 1.20; 95% *CI*, 1.12–1.29). Also, no literature was observed in the sensitivity analysis that had a significant impact on the study results, and no publication bias was observed.

Increasingly, studies have examined the relationship between HBV infection and the risk of CKD prevalence. Several previous meta-analyses have not observed a significant correlation between HBV infection and risk of CKD prevalence, with pooled effect estimates and their 95% *CIs* were 1.05 (0.56, 1.98) and 2.22 (0.95; 3.50), respectively [17, 48]. A recently published meta-analysis by Fabrizi F et al. found that HBV infection increased the risk of CKD (*OR*, 1.19; 95% *CI* 1.11–1.27) [11]. A recently published case-control study based on a Chinese population found that HBV infection promoted an increased risk of CKD (*OR*, 2.099; 95% *CI* 1.128–3.907) [14]. In this study, we found that HBV infection was associated with an increased risk of developing chronic kidney disease, which is consistent with the results of a recently published meta-analysis.

Unfortunately, our analysis found substantial heterogeneity in prior published studies (*I*^*2*^ = 85.7%, *P* < 0.001). In order to explore sources of heterogeneity, heterogeneity was assessed using meta-regression and subgroup analyses. However, study type, region, reference year, study outcome, and sample size were not sources of heterogeneity. Although studies providing adjusted outcome estimates (*HR*/*OR*) were included in our study, there may still be residual confounding factors. Therefore, sources of article heterogeneity could not be easily excluded. Meanwhile, because complete covariate information was not given across studies, we were unable to conduct a more comprehensive exploration of the sources of heterogeneity. For example, the specific inclusion and exclusion criteria for studies included in the literature may vary, which may account for the high degree of heterogeneity.

The mechanisms underlying the association between HBV and CKD development have not been fully elucidated. Nonetheless, the relationship between chronic HBV infection and kidney disease was reported in an article more than fifty years ago [49]. It has been suggested that the deposition of immune complexes in the kidney plays a key role in the pathogenesis of HBV-related nephropathy [50]. It is likely due to low molecular weight HBeAg (3 × 10^5^ Da) crossing the glomerular basement membrane to form subepithelial immune deposits, which leads to glomerular and interstitial tubular damage and contributes to the decline in renal function [51, 52]. Secondly, Deng et al. showed that excessive apoptosis of renal proximal tubular cells may also be associated with renal injury in patients with chronic HBV infection [53]. In addition, six nucleotide analogues (NAs) have been approved for the treatment of chronic HBV. Nevertheless, all NAs are excreted via the renal route and suffer from some degree of nephrotoxicity [54]. Therefore, dosing adjustments should be made according to the overall clinical status of chronic HBV infection to avoid causing renal impairment [55].

Our study has several advantages. Firstly, this study synthesizes several recently published large studies on the relationship between HBV infection and the risk of CKD, and provides more reliable evidence. Secondly, the study area included Asia, Europe, and the Americas, which can better represent the international research landscape. Generally, the results of our meta-analysis are similar to related articles recently published by other scholars.

Nevertheless, there are some limitations to this study. Firstly, the included studies contained a large proportion of case-control studies and cohort studies, which may be subject to selection bias and recall bias. Secondly, the inclusion of a large proportion of cross-sectional studies in this study made it difficult to establish a causal association between HBV infection and risk of CKD. Thirdly, our subgroup analysis could not explain the source of heterogeneity. In addition, although this study developed strict inclusion and exclusion criteria and used the NOS scale to assess the quality of the included articles during the screening process, there was still a degree of subjectivity in the assessment of the literature.

## Conclusions

In conclusion, this study found that HBV infection was associated with a significant increase in the risk of CKD. However, the current study still cannot directly determine this cause-and-effect relationship. Thus, more comprehensive prospective longitudinal studies are needed in the future to provide further exploration and explanation of the association between hepatitis B virus and the risk of developing chronic kidney disease.

### Electronic supplementary material

Below is the link to the electronic supplementary material.

Supplementary Material 1

## Data Availability

Data sharing is not applicable to this paper as no datasets were generated or analyzed for this study.

## Abbreviations

95% CI: 95% confidence intervals
CKD: Chronic kidney disease
CNKI: Chinese National Knowledge Infrastructure
ESRD: End-stage renal disease
GFR: Glomerular filtration rate
GN: Glomerulonephritis
HBV: Hepatitis B virus
HR: Hazard ratios
NAs: Nucleotide analogues
NOS: Newcastle-Ottawa Quality Scale
OR: Odds risks
PAN: Polyarteritis nodosa
