# Supplementary material for: Hepatitis B virus infection as a risk factor for chronic kidney disease: a systematic review and meta-analysis
Source: BMC Infect Dis. 2024 Jun 22;24:620. doi: 10.1186/s12879-024-09546-z (PMC11193185; doi:10.1186/s12879-024-09546-z)
Supplement: Supplementary file 1 — Supplementary Material 1 [file 12879_2024_9546_MOESM1_ESM.docx]

**Supplementary Material 1 Search terms for literature search within the seven databases to evaluate the association of HBV with CKD risk**

**Chinese National Knowledge Infrastructure (CNKI)**

(Hepatitis B virus infection + hepatitis B + viral hepatitis B virus + chronic hepatitis B + hepatitis B virus + HBV) AND (chronic kidney disease + Chronic renal insufficiency + Chronic renal disease + glomerulonephritis + tubular damage + renal vascular disease + liver disease + CKD + cystitis + kidney disease + chronic nephritis + chronic glomerulonephritis + Chronic renal failure)

**Wanfang**

(Subject Term:( Hepatitis B virus infection) or TI or AB:( Hepatitis B virus infection or hepatitis B or viral hepatitis B virus or chronic hepatitis B or hepatitis B virus or HBV)) and (Subject Term:( chronic kidney disease) or TI or AB: ( chronic kidney disease or Chronic renal insufficiency or Chronic renal disease or glomerulonephritis or tubular damage or renal vascular disease or liver disease or CKD or cystitis or kidney disease or chronic nephritis or chronic glomerulonephritis or Chronic renal failure))

**China Science and Technology Journal (VIP)**

(Hepatitis B virus infection or hepatitis B or viral hepatitis B virus or chronic hepatitis B or hepatitis B virus or HBV) AND (chronic kidney disease or Chronic renal insufficiency or Chronic renal disease or glomerulonephritis or tubular damage or renal vascular disease or liver disease or CKD or cystitis or kidney disease or chronic nephritis or chronic glomerulonephritis or Chronic renal failure)

**PubMed**

(((((((((((((((((((((((((("Hepatitis B virus"[Mesh]) OR (Hepatitis B virus[Title/Abstract])) OR (B virus, Hepatitis[Title/Abstract])) OR (Hepatitis B viruses[Title/Abstract])) OR (viruses, Hepatitis B[Title/Abstract])) OR (Hepatitis Virus, Homologous Serum[Title/Abstract])) OR (Dane Particle[Title/Abstract])) OR (Particle, Dane[Title/Abstract])) OR (Hepatitis B Virus Infection[Title/Abstract])) OR (Chronic Hepatitis B Virus Infection[Title/Abstract])) OR (Chronic Hepatitis B[Title/Abstract])) OR (Hepatitis B Virus Infection, Chronic[Title/Abstract])) OR (HBV[Title/Abstract])) OR (Hepatitis B[Title/Abstract])) OR (australia virus[Title/Abstract])) OR (Hepatitis virus B[Title/Abstract])) OR (serum hepatitis virus[Title/Abstract])) OR (hepatitis, serum[Title/Abstract]))) OR (hippie hepatitis[Title/Abstract])) OR (injection hepatitis[Title/Abstract])) OR (serum hepatitis[Title/Abstract])) OR (type b hepatitis[Title/Abstract])) OR (viral hepatitis type B[Title/Abstract])) OR (virus hepatitis type B'[Title/Abstract]) AND (((((((((((((((((((((((((((((((((((((((((((((((((((((("Renal Insufficiency, Chronic"[Mesh]) OR (Renal Insufficiency, Chronic[Title/Abstract])) OR (Chronic Renal Insufficiencies[Title/Abstract])) OR (Renal Insufficiencies, Chronic[Title/Abstract])) OR (Chronic Renal Insufficiency[Title/Abstract])) OR (Kidney Insufficiency, Chronic[Title/Abstract])) OR (Chronic Kidney Insufficiency[Title/Abstract])) OR (Chronic Kidney Insufficiencies[Title/Abstract])) OR (Kidney Insufficiencies, Chronic[Title/Abstract])) OR (Chronic Kidney Diseases[Title/Abstract])) OR (Chronic Kidney Disease[Title/Abstract])) OR (Disease, Chronic Kidney[Title/Abstract])) OR (Diseases, Chronic Kidney[Title/Abstract])) OR (Kidney Disease, Chronic[Title/Abstract])) OR (Kidney Diseases, Chronic[Title/Abstract])) OR (Chronic Renal Diseases[Title/Abstract])) OR (Chronic Renal Disease[Title/Abstract])) OR (Chronic Renal Disease[Title/Abstract])) OR (Diseases, Chronic Renal[Title/Abstract])) OR (Renal Disease, Chronic[Title/Abstract])) OR (Renal Diseases, Chronic[Title/Abstract])) OR (Kidney Diseases[Title/Abstract])) OR (Disease, Kidney[Title/Abstract])) OR (Diseases, Kidney[Title/Abstract])) OR (Kidney Disease[Title/Abstract])) OR (Kidney Failure, Chronic[Title/Abstract])) OR (End-Stage Kidney Disease[Title/Abstract])) OR (Disease, End-Stage Kidney[Title/Abstract])) OR (End Stage Kidney Disease[Title/Abstract])) OR (Kidney Disease, End-Stage[Title/Abstract])) OR (Chronic Kidney Failure[Title/Abstract])) OR (End-Stage Renal Disease[Title/Abstract])) OR (Disease, End-Stage Renal[Title/Abstract])) OR (End Stage Renal Disease[Title/Abstract])) OR (Renal Disease, End-Stage[Title/Abstract])) OR (Renal Disease, End Stage[Title/Abstract])) OR (Renal Failure, End-Stage[Title/Abstract])) OR (End-Stage Renal Failure[Title/Abstract])) OR (Renal Failure, End Stage[Title/Abstract])) OR (Renal Failure, Chronic[Title/Abstract])) OR (Chronic Renal Failure[Title/Abstract])) OR (ESRD[Title/Abstract])) OR (chronic kidney disorder[Title/Abstract])) OR (chronic nephropathy[Title/Abstract])) OR (chronic renal failure[Title/Abstract])) OR (kidney function, chronic disease[Title/Abstract])) OR (kidney disorder[Title/Abstract])) OR (kidney pathology[Title/Abstract])) OR (nephropathy[Title/Abstract])) OR (perirenal infection[Title/Abstract])) OR (perinephritis[Title/Abstract])) OR (renal disease[Title/Abstract])) OR (renal disorder[Title/Abstract])) OR (unilateral kidney disease[Title/Abstract])) Filters: from 1000/1/1 - 2023/3/20 Sort by: Most Recent

**Cochrane Library**

(Hepatitis B virus or B virus, Hepatitis or Hepatitis B viruses or viruses, Hepatitis B or Hepatitis Virus, Homologous Serum or Dane Particle or Particle, Dane or Hepatitis B Virus Infection or Chronic Hepatitis B Virus Infection or Chronic Hepatitis B or Hepatitis B Virus Infection, Chronic or HBV or Hepatitis B or australia virus or Hepatitis virus B or human hepatitis B virus or serum hepatitis virus or hepatitis, serum or hippie hepatitis or injection hepatitis or serum hepatitis or type b hepatitis or viral hepatitis type B) AND (Renal Insufficiency, Chronic or Chronic Renal Insufficiencies or Renal Insufficiencies, Chronic or Chronic Renal Insufficiency or Kidney Insufficiency, Chronic or Chronic Kidney Insufficiency or Chronic Kidney Insufficiencies or Kidney Insufficiencies, Chronic or Chronic Kidney Diseases or Chronic Kidney Disease or Disease, Chronic Kidney or Diseases, Chronic Kidney or Kidney Disease, Chronic or Kidney Diseases, Chronic or Chronic Renal Diseases or Chronic Renal Disease or Disease, Chronic Renal or Diseases, Chronic Renal or Renal Disease, Chronic or Renal Diseases, Chronic or Kidney Diseases or Disease, Kidney or Diseases, Kidney or Kidney Disease or Kidney Failure, Chronic or End-Stage Kidney Disease or Disease, End-Stage Kidney or End Stage Kidney Disease or Kidney Disease, End-Stage or Chronic Kidney Failure or End-Stage Renal Disease or Disease, End-Stage Renal or End Stage Renal Disease or Renal Disease, End-Stage or Renal Disease, End Stage or Renal Failure, End-Stage or End-Stage Renal Failure or Renal Failure, End Stage or Renal Failure, Chronic or Chronic Renal Failure or ESRD or chronic kidney disorder or chronic nephropathy or chronic renal failure or kidney function, chronic disease or kidney function, chronic disease or kidney disorder or kidney pathology or nephropathy or perirenal infection or perinephritis or renal disease or renal disorder or unilateral kidney disease)

**Web of Science**

(TS=(Hepatitis B virus) OR AB=(Hepatitis B virus OR B virus, Hepatitis OR Hepatitis B viruses OR viruses, Hepatitis B OR Hepatitis Virus, Homologous Serum OR Dane Particle OR Particle, Dane OR Hepatitis B Virus Infection OR Chronic Hepatitis B Virus Infection OR Chronic Hepatitis B OR Hepatitis B Virus Infection, Chronic OR HBV OR Hepatitis B OR australia virus OR Hepatitis virus B OR serum hepatitis virus OR hepatitis, serum OR hippie hepatitis OR injection hepatitis OR serum hepatitis OR type b hepatitis OR viral hepatitis type B OR virus hepatitis type B) )AND (TS=(Renal Insufficiency, Chronic) OR AB=(Renal Insufficiency, Chronic OR Chronic Renal Insufficiencies OR Renal Insufficiencies, Chronic OR Chronic Renal Insufficiency OR Kidney Insufficiency, Chronic OR Chronic Kidney Insufficiency OR Chronic Kidney Insufficiencies OR Kidney Insufficiencies, Chronic OR Chronic Kidney Diseases OR Chronic Kidney Disease OR Disease, Chronic Kidney OR Diseases, Chronic Kidney OR Kidney Disease, Chronic OR Kidney Diseases, Chronic OR Chronic Renal Diseases OR Chronic Renal Disease OR Chronic Renal Disease OR Diseases, Chronic Renal OR Renal Disease, Chronic OR Renal Diseases, Chronic OR Kidney Diseases OR Disease, Kidney OR Diseases, Kidney OR Kidney Disease OR Kidney Failure, Chronic OR End-Stage Kidney Disease OR Disease, End-Stage Kidney OR End Stage Kidney Disease OR Kidney Disease, End-Stage OR Chronic Kidney Failure OR End-Stage Renal Disease OR Disease, End-Stage Renal OR End Stage Renal Disease OR Renal Disease, End-Stage OR Renal Disease, End Stage OR Renal Failure, End-Stage OR End-Stage Renal Failure OR Renal Failure, End Stage OR Renal Failure, Chronic OR Chronic Renal Failure OR ESRD OR chronic kidney disorder OR chronic nephropathy OR chronic renal failure OR kidney function, chronic disease OR kidney disorder OR kidney pathology OR nephropathy OR perirenal infection OR perinephritis OR renal disease OR renal disorder OR unilateral kidney disease) )

**Embase**

(Hepatitis B virus OR B virus, Hepatitis OR Hepatitis B viruses OR viruses, Hepatitis B OR Hepatitis Virus, Homologous Serum OR Dane Particle OR Particle, Dane OR Hepatitis B Virus Infection OR Chronic Hepatitis B Virus Infection OR Chronic Hepatitis B OR Hepatitis B Virus Infection, Chronic OR HBV OR Hepatitis B OR australia virus OR Hepatitis virus B OR serum hepatitis virus OR hepatitis, serum OR hippie hepatitis OR injection hepatitis OR serum hepatitis OR type b hepatitis OR viral hepatitis type B OR virus hepatitis type B) AND (Renal Insufficiency, Chronic OR Chronic Renal Insufficiencies OR Renal Insufficiencies, Chronic OR Chronic Renal Insufficiency OR Kidney Insufficiency, Chronic OR Chronic Kidney Insufficiency OR Chronic Kidney Insufficiencies OR Kidney Insufficiencies, Chronic OR Chronic Kidney Diseases OR Chronic Kidney Disease OR Disease, Chronic Kidney OR Diseases, Chronic Kidney OR Kidney Disease, Chronic OR Kidney Diseases, Chronic OR Chronic Renal Diseases OR Chronic Renal Disease OR Chronic Renal Disease OR Diseases, Chronic Renal OR Renal Disease, Chronic OR Renal Diseases, Chronic OR Kidney Diseases OR Disease, Kidney OR Diseases, Kidney OR Kidney Disease OR Kidney Failure, Chronic OR End-Stage Kidney Disease OR Disease, End-Stage Kidney OR End Stage Kidney Disease OR Kidney Disease, End-Stage OR Chronic Kidney Failure OR End-Stage Renal Disease OR Disease, End-Stage Renal OR End Stage Renal Disease OR Renal Disease, End-Stage OR Renal Disease, End Stage OR Renal Failure, End-Stage OR End-Stage Renal Failure OR Renal Failure, End Stage OR Renal Failure, Chronic OR Chronic Renal Failure OR ESRD OR chronic kidney disorder OR chronic nephropathy OR chronic renal failure OR kidney function, chronic disease OR kidney disorder OR kidney pathology OR nephropathy OR perirenal infection OR perinephritis OR renal disease OR renal disorder OR unilateral kidney disease)

**Supplementary Table 1 Quality assessment of the case-control studies and cohorts studies included in the meta-analysis according to the Newcastle-Ottawa scale**

| **Author** | **Year of publication** | **Study type** | | **Selection** | | | | | | | **Comparability** | | **Exposure/Outcome** | | | | | **Total score** | |  |
| --- | --- | --- | --- | --- | --- | --- | --- | --- | --- | --- | --- | --- | --- | --- | --- | --- | --- | --- | --- | --- |
|  |  |  |  | **Adequate definition of cases** | | **Representativeness of cases** | | **Selesction of controls** | | **Definition of controls** | **Control for important factor or additional factor** | | **Ascertainment of exposure** | | **Same method of ascertainment for cases and controls** | | **Non-response rate** |  |  |  |
| Kim SE | 2018 | Case-control | | 1 | | 1 | | 0 | | 1 | 2 | | 1 | | 1 | | 1 | 9 | |  |
| Liu Y | 2021 | Case-control | | 1 | | 1 | | 0 | | 1 | 2 | | 1 | | 1 | | 1 | 9 | |  |
| Su SL | 2015 | Case-control | | 1 | | 1 | | 0 | | 1 | 0 | | 1 | | 1 | | 1 | 7 | |  |
| Vu V | 2019 | Case-control | | 1 | | 1 | | 0 | | 1 | 2 | | 1 | | 1 | | 1 | 9 | |  |
|  |  |  | | **Representativeness of the exposed cohort** | | **Selection of the nonexposed cohort** | | **Ascertainment of exposure** | | **Demonstration that the outcome of interest was not present at the start of the study** | **Control for important factor or additional factor** | | **Assessment of outcome** | | **Select an adequate follow-up period for the outcome of interset** | | **Adequacy of follow-up of cohort** |  | |  |
| Chen YC | 2015 | Cohort | | 1 | | 1 | | 1 | | 0 | 2 | | 1 | | 1 | | 0 | 7 | |  |
| Du Y | 2019 | Cohort | | 1 | | 1 | | 1 | | 1 | 1 | | 1 | | 0 | | 1 | 7 | |  |
| Geng XX | 2020 | Cohort | | 0 | | 1 | | 1 | | 0 | 2 | | 1 | | 0 | | 0 | 5 | |  |
| Hong Y | 2018 | Cohort | | 0 | | 1 | | 1 | | 1 | 1 | | 1 | | 1 | | 0 | 6 | |  |
| Kong XL | 2016 | Cohort | | 0 | | 1 | | 1 | | 1 | 1 | | 1 | | 1 | | 1 | 7 | |  |
| Mocroft A | 2012 | Cohort | | 0 | | 1 | | 1 | | 1 | 1 | | 1 | | 1 | | 0 | 6 | |  |
| Si J | 2018 | Cohort | | 1 | | 1 | | 1 | | 1 | 1 | | 1 | | 1 | | 1 | 8 | |  |
| Cheng AY | 2006 | Cohort | | 0 | | 1 | | 1 | | 1 | 1 | | 1 | | 1 | | 0 | 6 | |  |
| Fang J | 2018 | | Cohort | | 1 | | 1 | | 1 | 0 | | 1 | | 1 | | 0 | 1 | | 6 | |
| Hwang JC | 2016 | | Cohort | | 0 | | 1 | | 1 | 1 | | 2 | | 1 | | 1 | 0 | | 7 | |
| Lai TS | 2017 | | Cohort | | 1 | | 1 | | 1 | 1 | | 1 | | 1 | | 1 | 1 | | 8 | |
| Lee JJ | 2014 | | Cohort | | 0 | | 1 | | 1 | 1 | | 1 | | 1 | | 1 | 0 | | 6 | |
| Nguyen MH | 2019 | | Cohort | | 1 | | 1 | | 1 | 1 | | 2 | | 1 | | 1 | 1 | | 9 | |
| Chen YC | 2018 | | Cohort | | 1 | | 1 | | 1 | 1 | | 2 | | 1 | | 1 | 0 | | 8 | |
| Lo MK | 2004 | | Cohort | | 0 | | 1 | | 1 | 1 | | 1 | | 1 | | 1 | 0 | | 6 | |
| Tartof SY | 2018 | | Cohort | | 1 | | 1 | | 1 | 1 | | 1 | | 1 | | 1 | 1 | | 8 | |

**Supplementary Table 2 Quality assessment of the cross-sectional studies included in the meta-analysis according to the adapt Newcastle-Ottawa scale**

| **Author** | **Year of publication** | **Study type** | **Selection** | | | | **Comparability** | **Exposure/Outcome** | | **Total score** |
| --- | --- | --- | --- | --- | --- | --- | --- | --- | --- | --- |
|  |  |  | **Representativeness of the sample** | **Sample size** | **Non-respondents** | **Ascertainment of the exposure (risk factor)** | **Control for important factor or additional factor** | **Ascertainment of exposure** | **Statistical test** |  |
| Cai J | 2012 | Cross-sectional | 1 | 1 | 0 | 2 | 2 | 2 | 1 | 9 |
| Ishizaka N | 2008 | Cross-sectional | 0 | 1 | 0 | 2 | 2 | 2 | 1 | 8 |
| Lee JJ | 2010 | Cross-sectional | 0 | 1 | 1 | 2 | 2 | 2 | 1 | 9 |
| Lin MY | 2012 | Cross-sectional | 1 | 1 | 0 | 2 | 2 | 2 | 1 | 9 |
| Lin S | 2020 | Cross-sectional | 0 | 1 | 0 | 2 | 2 | 2 | 1 | 8 |
| Senghore T | 2013 | Cross-sectional | 1 | 1 | 1 | 1 | 1 | 1 | 1 | 7 |
| Zeng Q | 2014 | Cross-sectional | 0 | 1 | 0 | 2 | 2 | 2 | 1 | 8 |
| Zhang H | 2019 | Cross-sectional | 1 | 1 | 0 | 2 | 0 | 2 | 1 | 7 |
| Zhang L | 2008 | Cross-sectional | 1 | 1 | 1 | 2 | 1 | 2 | 1 | 9 |
| Du Y | 2017 | Cross-sectional | 1 | 1 | 1 | 2 | 1 | 2 | 1 | 9 |
| Huang JF | 2006 | Cross-sectional | 1 | 1 | 1 | 1 | 1 | 1 | 1 | 7 |


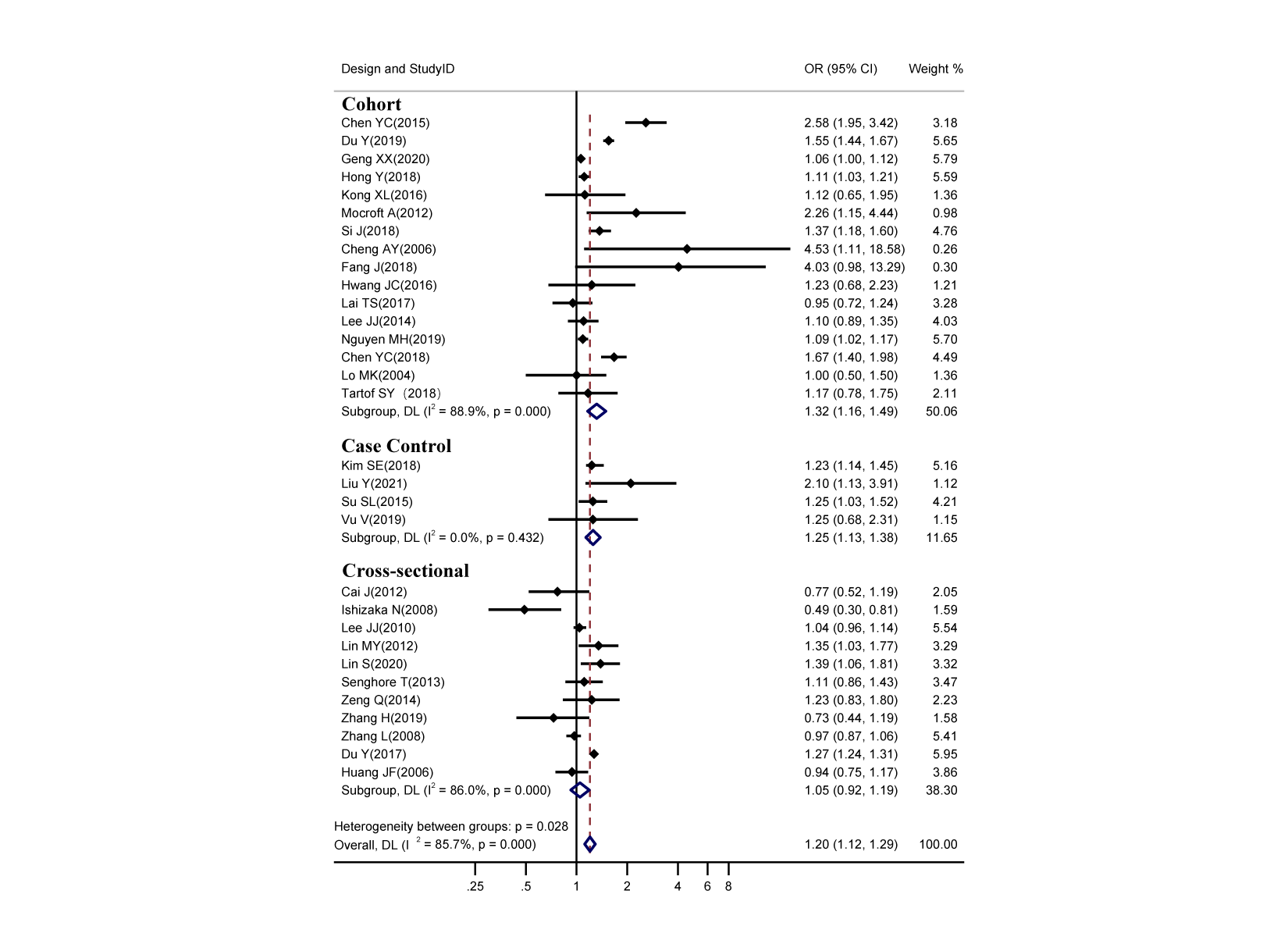


**Supplementary Figure 1 Forest plot for subgroup analysis: type of study**

**
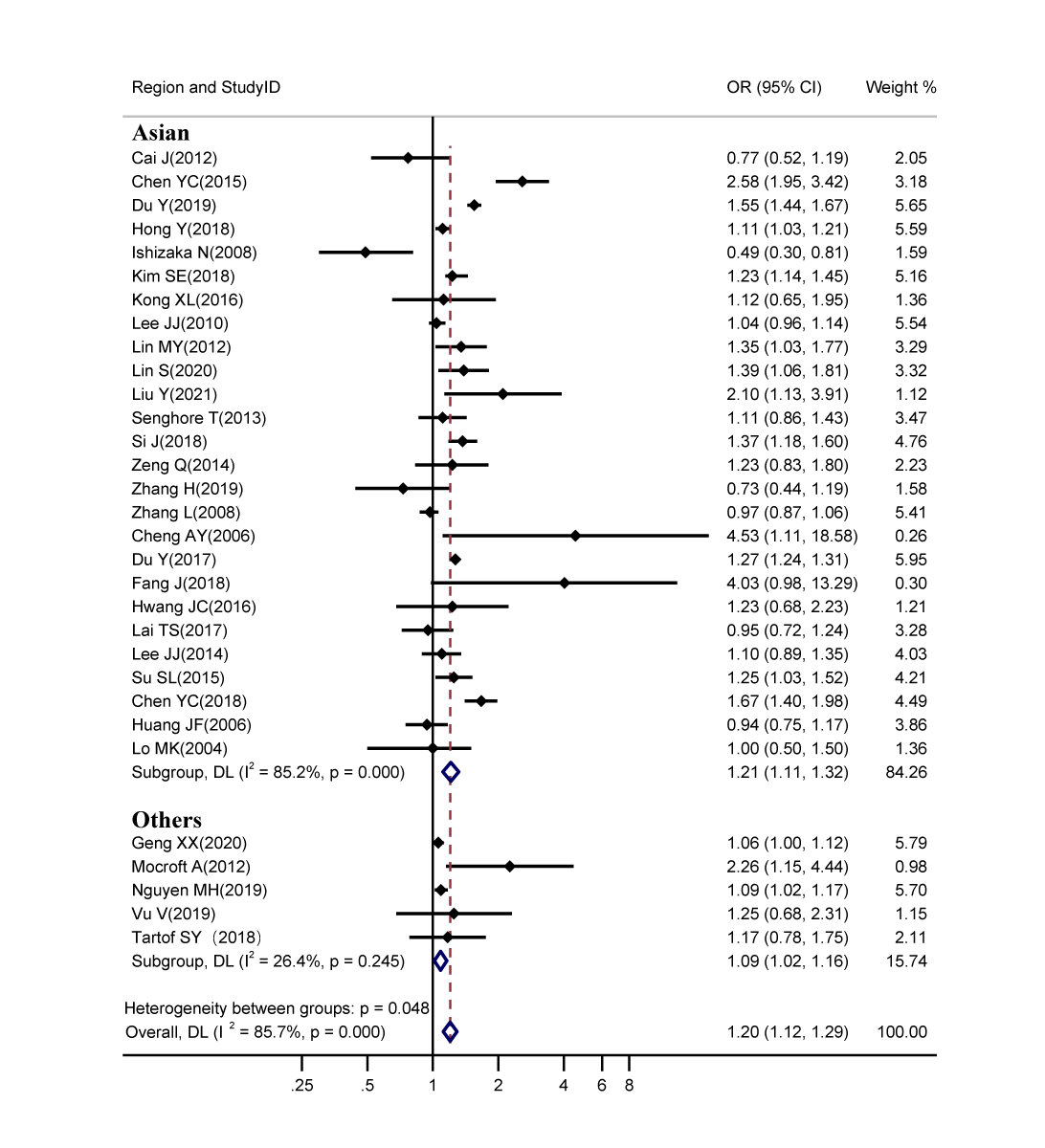
**

**Supplementary Figure 2 Forest plot for subgroup analysis: region**

**
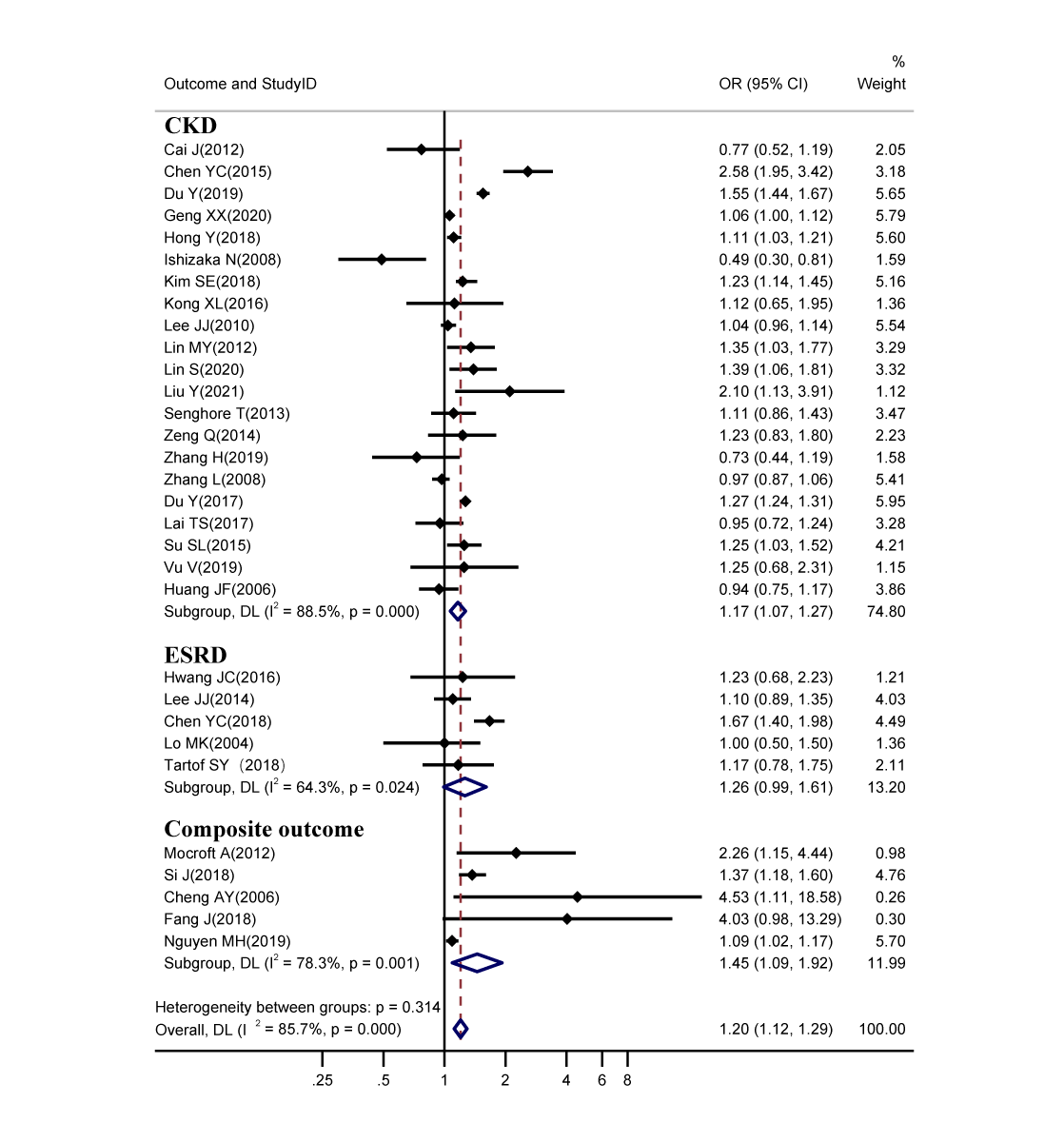
**

**Supplementary Figure 3 Forest plot for subgroup analysis: study of outcome**

**
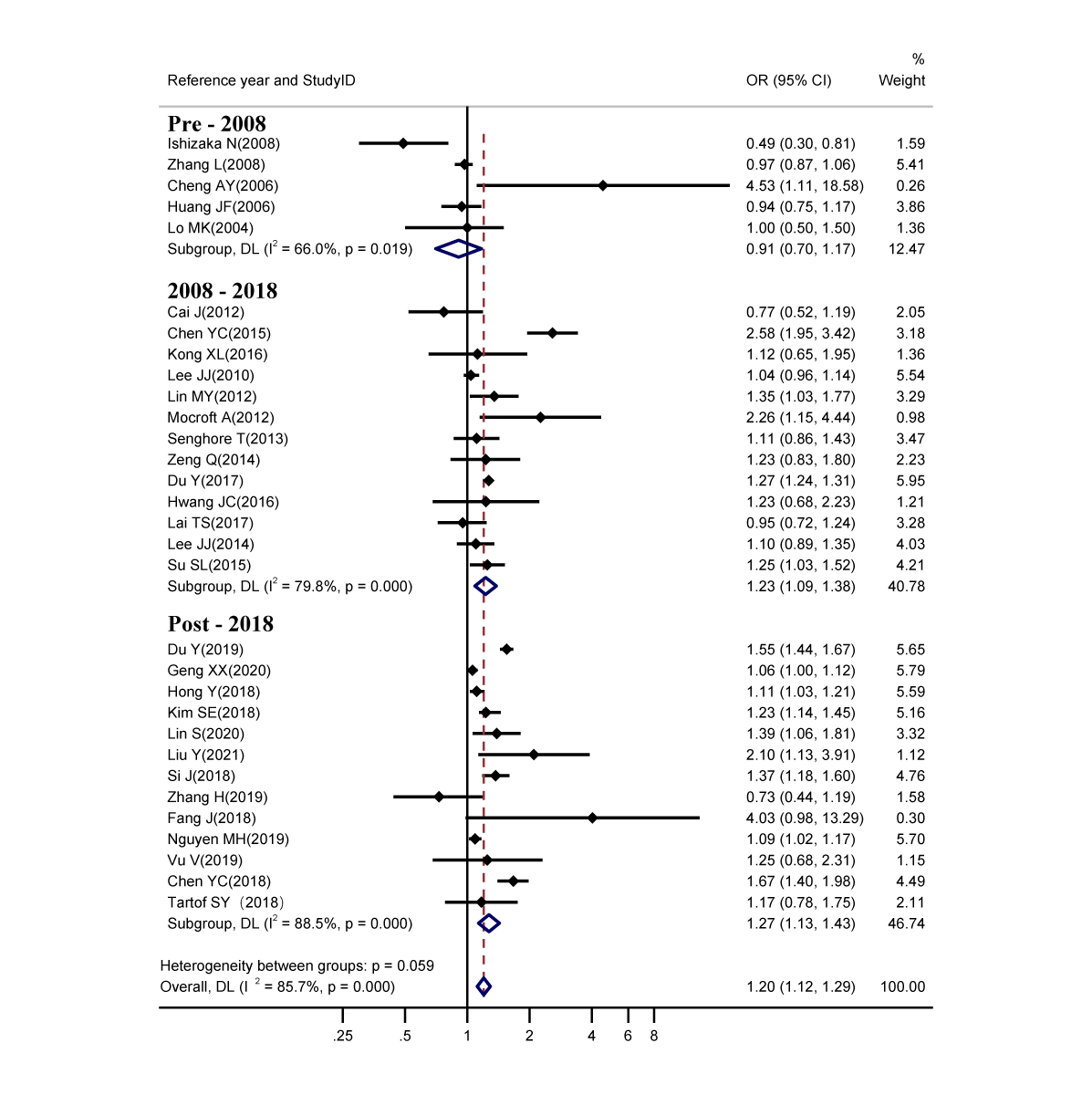
**

**Supplementary Figure 4 Forest plot for subgroup analysis: reference year**

**
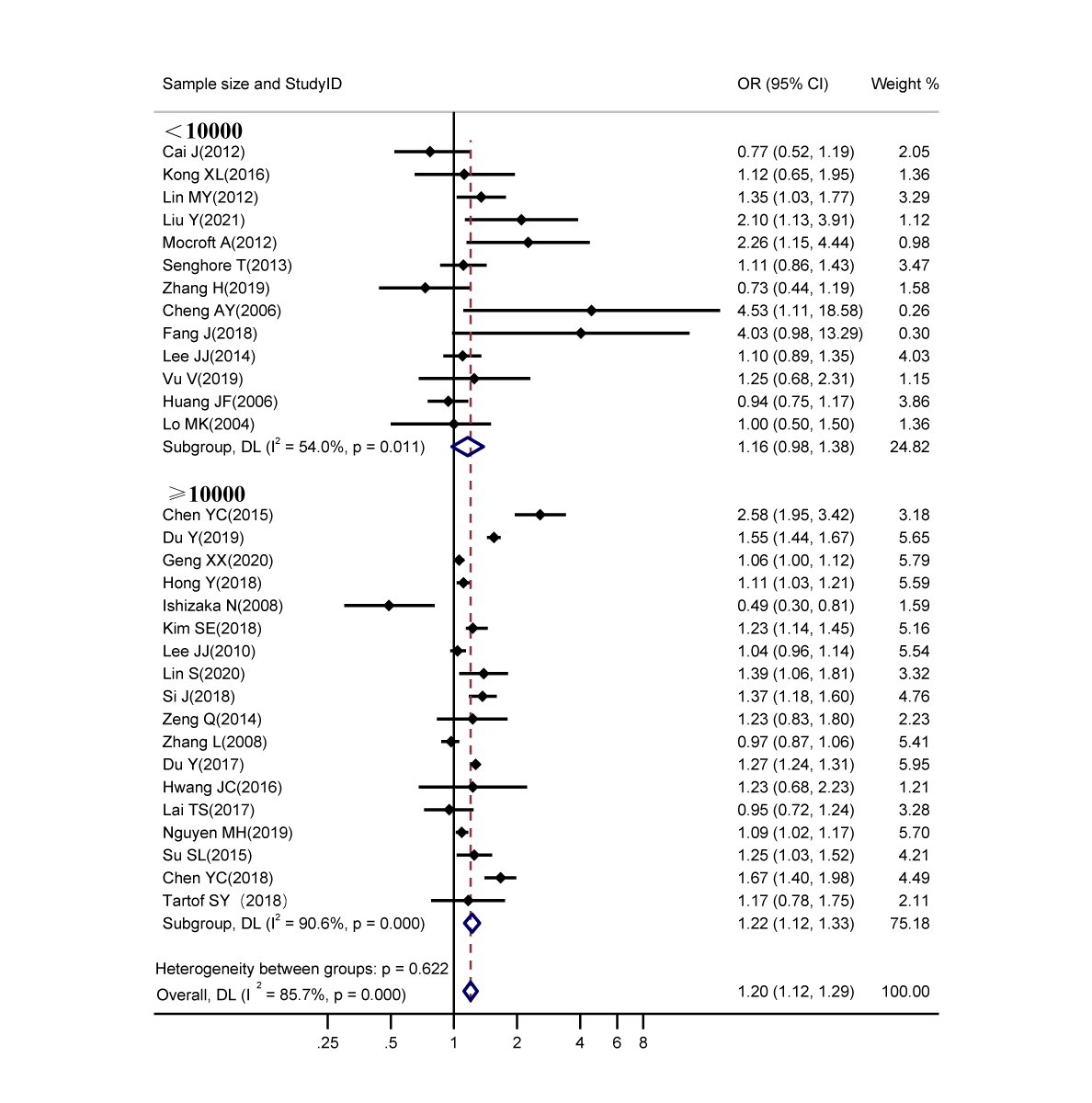
**

**Supplementary Figure 5 Forest plot for subgroup analysis: sample size**
